# Genome Wide Identification of SARS-CoV Susceptibility Loci Using the Collaborative Cross

**DOI:** 10.1371/journal.pgen.1005504

**Published:** 2015-10-09

**Authors:** Lisa E. Gralinski, Martin T. Ferris, David L. Aylor, Alan C. Whitmore, Richard Green, Matthew B. Frieman, Damon Deming, Vineet D. Menachery, Darla R. Miller, Ryan J. Buus, Timothy A. Bell, Gary A. Churchill, David W. Threadgill, Michael G. Katze, Leonard McMillan, William Valdar, Mark T. Heise, Fernando Pardo-Manuel de Villena, Ralph S. Baric

**Affiliations:** 1 Department of Epidemiology, University of North Carolina, Chapel Hill, Chapel Hill, North Carolina, United States of America; 2 Department of Genetics, University of North Carolina, Chapel Hill, Chapel Hill, North Carolina, United States of America; 3 Department of Microbiology, University of Washington, Seattle, Washington, United States of America; 4 Lineberger Comprehensive Cancer Center, University of North Carolina, Chapel Hill, Chapel Hill, North Carolina, United States of America; 5 The Jackson Laboratory, Bar Harbor, Maine, United States of America; 6 Department of Veterinary Pathobiology, Texas A&M University, College Station, Texas, United States of America; 7 Department of Computer Science, University of North Carolina, Chapel Hill, Chapel Hill, North Carolina, United States of America; Institut Pasteur; URA CNRS 2578, FRANCE

## Abstract

New systems genetics approaches are needed to rapidly identify host genes and genetic networks that regulate complex disease outcomes. Using genetically diverse animals from incipient lines of the Collaborative Cross mouse panel, we demonstrate a greatly expanded range of phenotypes relative to classical mouse models of SARS-CoV infection including lung pathology, weight loss and viral titer. Genetic mapping revealed several loci contributing to differential disease responses, including an 8.5Mb locus associated with vascular cuffing on chromosome 3 that contained 23 genes and 13 noncoding RNAs. Integrating phenotypic and genetic data narrowed this region to a single gene, *Trim55*, an E3 ubiquitin ligase with a role in muscle fiber maintenance. Lung pathology and transcriptomic data from mice genetically deficient in *Trim55* were used to validate its role in SARS-CoV-induced vascular cuffing and inflammation. These data establish the Collaborative Cross platform as a powerful genetic resource for uncovering genetic contributions of complex traits in microbial disease severity, inflammation and virus replication in models of outbred populations.

## Author Summary

New emerging pathogens are a significant threat to human health with at least six highly pathogenic viruses, including four respiratory viruses, having spread from animal hosts into the human population within the past 15 years. With the emergence of new pathogens, new and better animal models are needed in order to better understand the disease these pathogens cause; to assist in the rapid development of therapeutics; and importantly to evaluate the role of natural host genetic variation in regulating disease outcome. We used incipient lines of the Collaborative Cross, a newly available recombinant inbred mouse panel, to identify polymorphic host genes that contribute to SARS-CoV pathogenesis. We discovered new animal models that better capture the range of disease found in human SARS patients and also found four novel susceptibility loci governing various aspects of SARS-induced pathogenesis. By integrating statistical, genetic and bioinformatic approaches we were able to narrow candidate genome regions to highly likely candidate genes. We narrowed one locus to a single candidate gene, *Trim55*, and confirmed its role in the inflammatory response to SARS-CoV infection through the use of knockout mice. This work identifies a novel function for *Trim55* and also demonstrates the utility of the CC as a platform for identifying the genetic contributions of complex traits.

## Introduction

Severe Acute Respiratory Coronavirus (SARS-CoV) emerged in humans in Southeast Asia in 2002 and 2003 after evolving from related coronaviruses circulating in bats [1,2]. SARS-CoV caused an atypical pneumonia that was fatal in 10% of all patients and 50% of elderly patients [3,4]. Patients infected with SARS-CoV experienced fever, difficulty breathing and low blood oxygen saturation levels [5,6]. Severe cases developed diffuse alveolar damage (DAD) and acute respiratory distress syndrome (ARDS) and disease severity was positively associated with increased age [7]. Host genetic background is also thought to influence disease severity but this understanding is complicated by inconsistent sample collection, varying treatment regimens and the limited scope of the SARS epidemic in humans [3,8,9]. Existing animal models of SARS-CoV infection have revealed that this lethal pulmonary infection causes a denuding bronchiolitis and severe pneumonia which oftentimes progresses to acute respiratory failure [10,11,12]. More recently, a second emerging coronavirus designated Middle East Respiratory Coronavirus (MERS-CoV) emerged from bat and camel populations [13,14,15], and has caused ~38% mortality. Given the complex interplay between environmental, viral and host genetic variation in driving viral disease severity, as well as the difficulty of studying those factors in episodic outbreaks of pathogens such as SARS-CoV, MERS-CoV and other highly virulent zoonotic pathogens that cross the species barrier at regular intervals, novel approaches are needed to understand and identify those factors contributing to these diseases.

Host genetics play a critical role in regulating microbial disease severity, evidenced by the identification of highly penetrant host susceptibility alleles within *CCR5*, *FUT2*, *IL-28B* in controlling HIV, norovirus and HCV infection and disease severity, respectively [16,17,18]. However, most microbial infections cause complex disease phenotypes that are regulated by the interactions of oligogenic traits with reduced penetrance, making them extremely difficult to identify and validate in human populations during outbreaks. Mannose binding lectin (MBL) polymorphisms were alternatively associated with successful recovery from SARS-CoV infection and a poor outcome of infection [19,20], reflecting the complexity of performing candidate gene or genome wide association studies with limited human samples. The generation of a mouse adapted strain of SARS-CoV, MA15, allowed for development of a small animal model that replicated both human lung disease and the age-dependency of SARS-CoV pathogenesis [10]. MA15 infection of inbred mice deficient in various immune genes has greatly contributed to our understanding of the host response to SARS-CoV infection [21,22]. However, such studies have focused on extreme abrogation of rationally selected candidate genes and have not evaluated the role of undescribed polymorphisms in genes in a model mimicking the genetic diversity seen in the human population. As a complement to human genome wide association studies, here we apply a new approach designed to dissect the identity and contributions of monogenic and oligogenic variants on multiple traits in complex disease outcomes following acute virus infection in a mouse model of human populations.

The Collaborative Cross (CC), a novel eight-way recombinant inbred (RI) mouse strain panel, has recently become available to the scientific community [23,24,25]. The power of the CC for genetic mapping is enhanced by availability of complete genome sequences of the founder strains and rich bioinformatics resources [26,27,28]. The eight founder strains used to generate the CC (A/J, C57BL/6J, 129S1/SvImJ, NOD/ShiLtJ, NZO/HILtJ, CAST/EiJ, PWK/PhJ and WSB/EiJ) are phenotypically diverse and capture single nucleotide polymorphisms (SNPs) and insertion/deletions (In/Dels) at approximately twice the frequency of common variants in human populations [24,29,30,31,32]. The derivation of CC strains from these multiple founders has proven to be useful for identifying polymorphisms that are responsible for a variety of traits [23]. The CC supports precise genetic mapping and, because the CC strains are genetically reproducible, it also serves as a robust validation platform and reference resource for integrative systems genetics applications.

Here, we studied incipient lines of the CC (the preCC) to identify host genes that contributed to SARS-CoV MA15 infection and pathogenesis. We identified four novel quantitative trail loci (QTLs) contributing to SARS-CoV pathogenesis. Within the *HrS1* QTL, a combination of approaches applied to the CC platform predicted a single gene candidate, *Trim55*, as the principle regulator of vascular cuffing after infection. Vascular cuffing is a commonly reported phenotype observed in response to a variety of insults including chemical injury and infection ([33,34,35]; high levels of vascular cuffing have been observed in models of severely pathogenic SARS-CoV infection [21,22]. Fluid vascular cuffing has been reported to decrease lug compliance suggesting an important physiologic consequence of this response [36]. Using knockout mice, we confirmed the role of *Trim55* in immune cell infiltration, demonstrating the utility of the CC platform for identifying single gene candidates that likely regulate novel immune functions in trans-endothelial migration and perivascular cuffing following virus infection.

## Results

### Expansion of SARS-CoV associated phenotypes across the genetically diverse preCC

**Fig 1:**
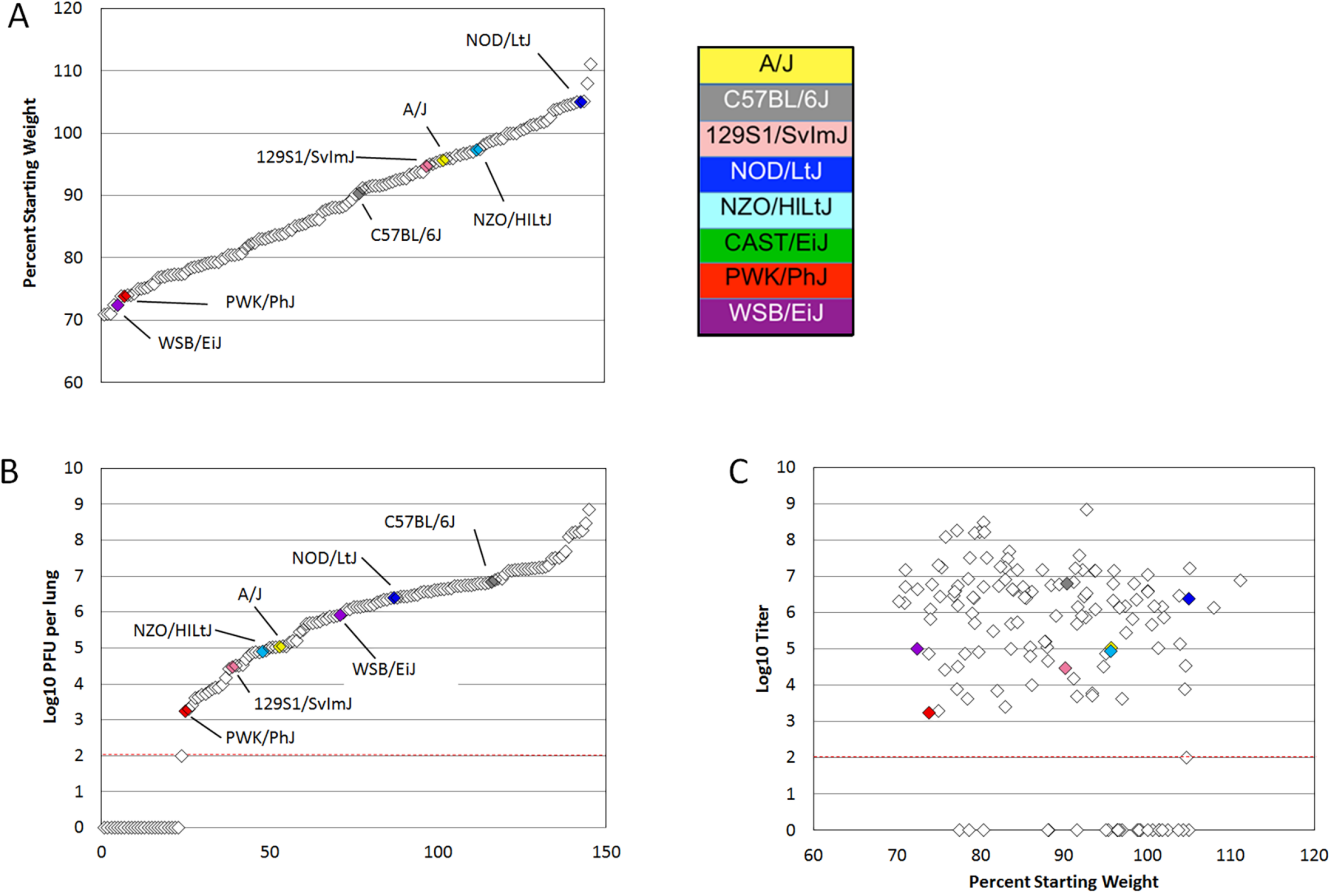
PreCC and founder strain phenotypes. (A) Weight loss is shown as percent of starting weight at day four post infection, individual preCC mice are shown in open diamonds and mean values for founders are shown in color. All CAST/EiJ mice died or were humanely euthanized before day four post infection. (B) Log transformed lung titer at day four post infection in individual preCC mice and founders, dashed line indicates the limit of detection at 100 PFU per lung. Individual preCC mice are shown in open diamonds, mean values for the founders are color coded by strain. (C) Lung titer vs. weight loss at day four post infection. Individual preCC mice are indicated in open diamonds, mean values for the founder strains are shown in color. The dashed line indicates the limit of detection at 100 PFU per lung.

Mice from the eight founder strains as well as 147 eight to twenty week old female preCC mice were infected with 10^5^ plaque forming units (PFU) of mouse adapted SARS-CoV, designated MA15 [10], and weight loss was observed over the course of a four day infection. At day four post infection mice were euthanized and tissue collected for assessment of viral load in the lung as well as virus-induced inflammation and pathology. A wide range of susceptibilities to SARS-CoV infection was found among the founder strains of the CC and the overall heritability of weight changes following SARS-CoV infection determined to have a coefficient of genetic determination of 0.72. NOD/ShiLtJ mice were resistant to infection and gained weight over the course of the experiment (**Figs 1A and S1A**). A/J, C57BL/6J,129S1/SvImJ and NZO/HILtJ mice experienced moderate and transient weight loss as previously described [21,22] while CAST/EiJ, PWK/PhJ and WSB/EiJ mice demonstrated extreme susceptibility to SARS-CoV infection including substantial weight loss and death (**Figs 1A, S1A and S1B**). Subsequent dose response studies using the three highly susceptible wild-derived strains indicated an LD_50_ of between 100 and 500 PFU for CAST/EiJ mice, between 500 PFU and 1000 PFU for PWK/PhJ and between 10^3^ and 10^5^ PFU for WSB/EiJ mice (**S1 Table**). PreCC mice infected with SARS-CoV ranged from over 30% weight loss by day four post infection to over 10% weight gain (**Fig 1A**), exceeding the range of susceptibilities observed in the founder strains. Additionally, 26 preCC mice (18% of the preCC cohort) succumbed to infection prior to the day four harvest point indicating extreme susceptibility to SARS-CoV infection.

Viral load in the lung at day four post infection was determined for each surviving preCC mouse as well as for each of the founder strains. Viral lung titers showed a heritability of 0.60 as measured by the coefficient of genetic determination amongst the 7 surviving founder strains. Amongst the founder strains, PWK/PhJ mice had the lowest viral loads in the lungs, with 1.75x10^3^ PFU per lung at day four post infection (**Figs 1B and S1B**). PWK/PhJ mice also showed significant weight loss and a low LD_50_ indicating that viral load was unlikely to be responsible for pathogenesis in these mice. In contrast, C57BL/6J mice had the highest amount of virus at 6.35x10^6^ PFU per lung. Lung titers in the preCC mice ranged from below the limit of detection (100 PFU/lung) to over 10^8^ PFU per lung, greatly exceeding the range of viral loads in the founder strains. Some preCC mice had viral loads in the lung below the 100 PFU limit of detection, despite having substantial weight loss. CAST/EiJ mice are extremely susceptible to SARS-CoV infection and do not survive until the day four post infection timepoint. **Fig 1C** shows the relationship between weight loss and lung titer at day four post infection. We found no correlation between viral load in the lung at day four post infection and weight loss (*r* = -0.014, p = 0.8938) when excluding those animals with viral loads below the limit of detection. When those animals were included in the analysis there is a significant, but not very explanatory correlation (*r* = -0.347, p = 0.00019) between the two phenotypes.

**Fig 2:**
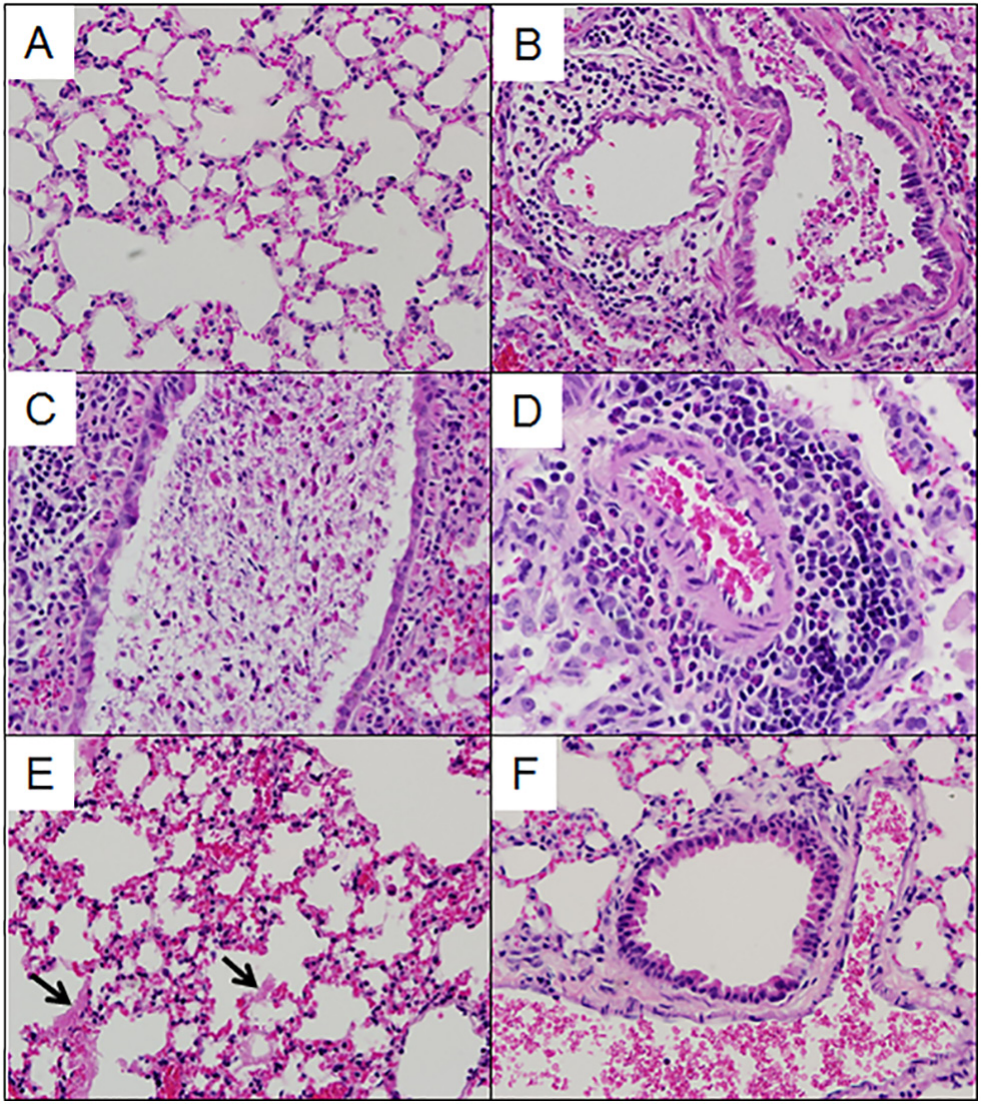
Lung pathology in select preCC mice. (A). OR63f51—normal parenchyma. (B). OR181f61—airway debris and cuffing and edema surrounding the associated vasculature. (C) OR220f57—denuded airway blocked with debris. (D) OR380f64 –perivascular cuffing including eosinophilia. (E) OR941f69 –alveolitis including hyaline membrane formation, arrows point to hyaline membranes. (F) OR5030f128 –normal airway and associated vasculature.

**Fig 3:**
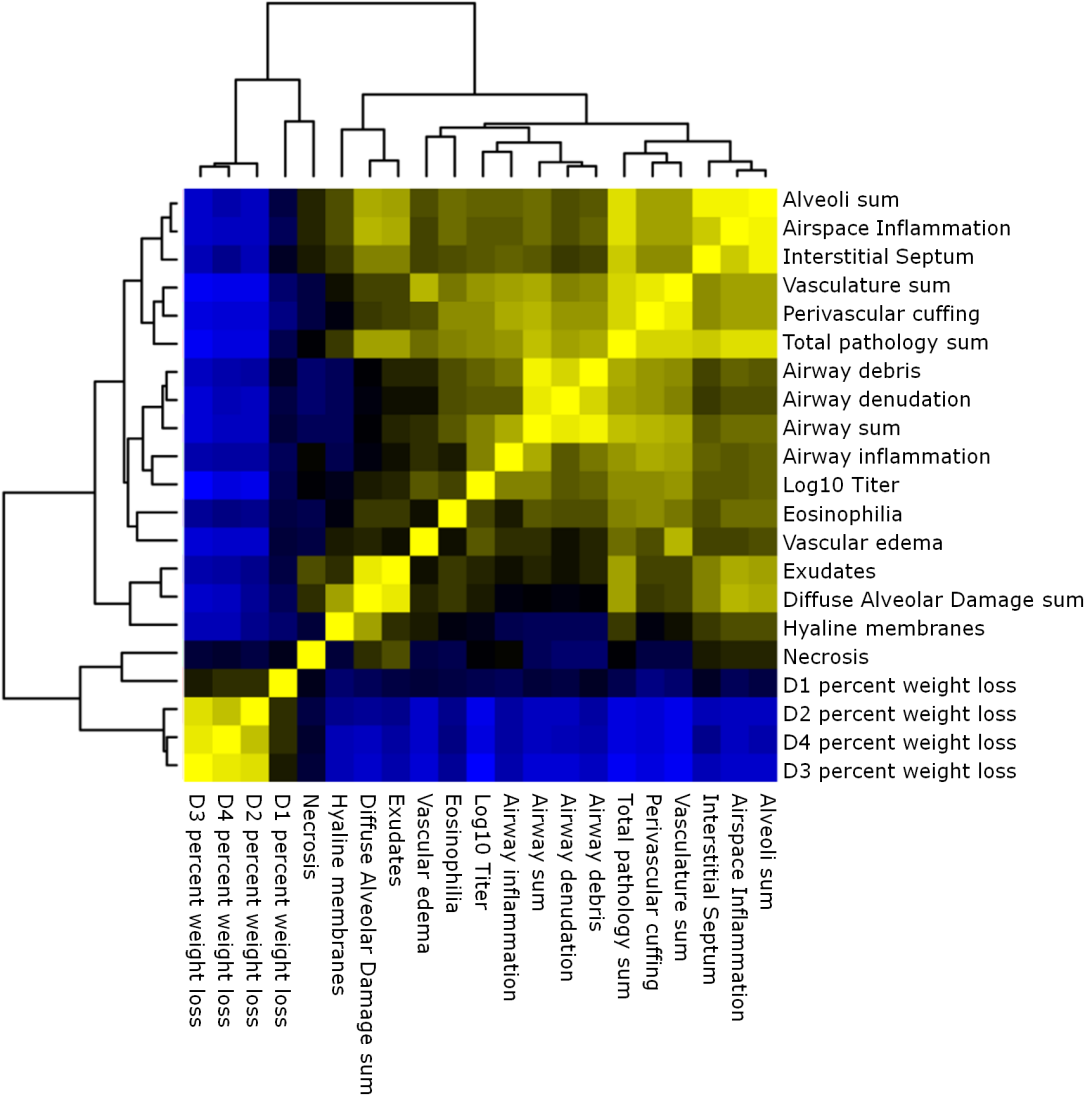
Phenotypic relationships. Heat map of the relationships between lung pathology, titer and weight loss in the preCC. Yellow indicates positive correlation and blue indicates negative correlation.

Multiple aspects of lung pathology were assessed in surviving preCC animals including disease and immune infiltrates in the airways, vasculature, alveoli and parenchyma and signs of DAD (**S2 Table)**. A wide variety of lung pathologies were found across the preCC mice including denudation of airway epithelial cells, airway debris, eosinophilia, hyaline membrane formation and vascular cuffing (**Fig 2A–2F**). Quantification of the overall pathology score along with select data ranges are shown in **S2 Fig**. Hyaline membrane formation and pulmonary edema with accompanying inflammation in the alveoli was a hallmark of SARS-CoV infection in human cases and is also evident in aged mouse models of disease [11]. In contrast to young founder strain animals, robust hyaline membrane formation was observed in 13% of preCC mice at day four post-infection, demonstrating that improved animal models are one likely outcome of infection studies in the CC. Phenotypic correlations of varying strengths were observed between aspects of lung pathology, inflammation, viral load at day four post infection, as well as weight loss across the course of the study (**Fig 3**).

### Multiple genetic loci contribute to aspects of SARS-CoV pathogenesis

**Fig 4:**
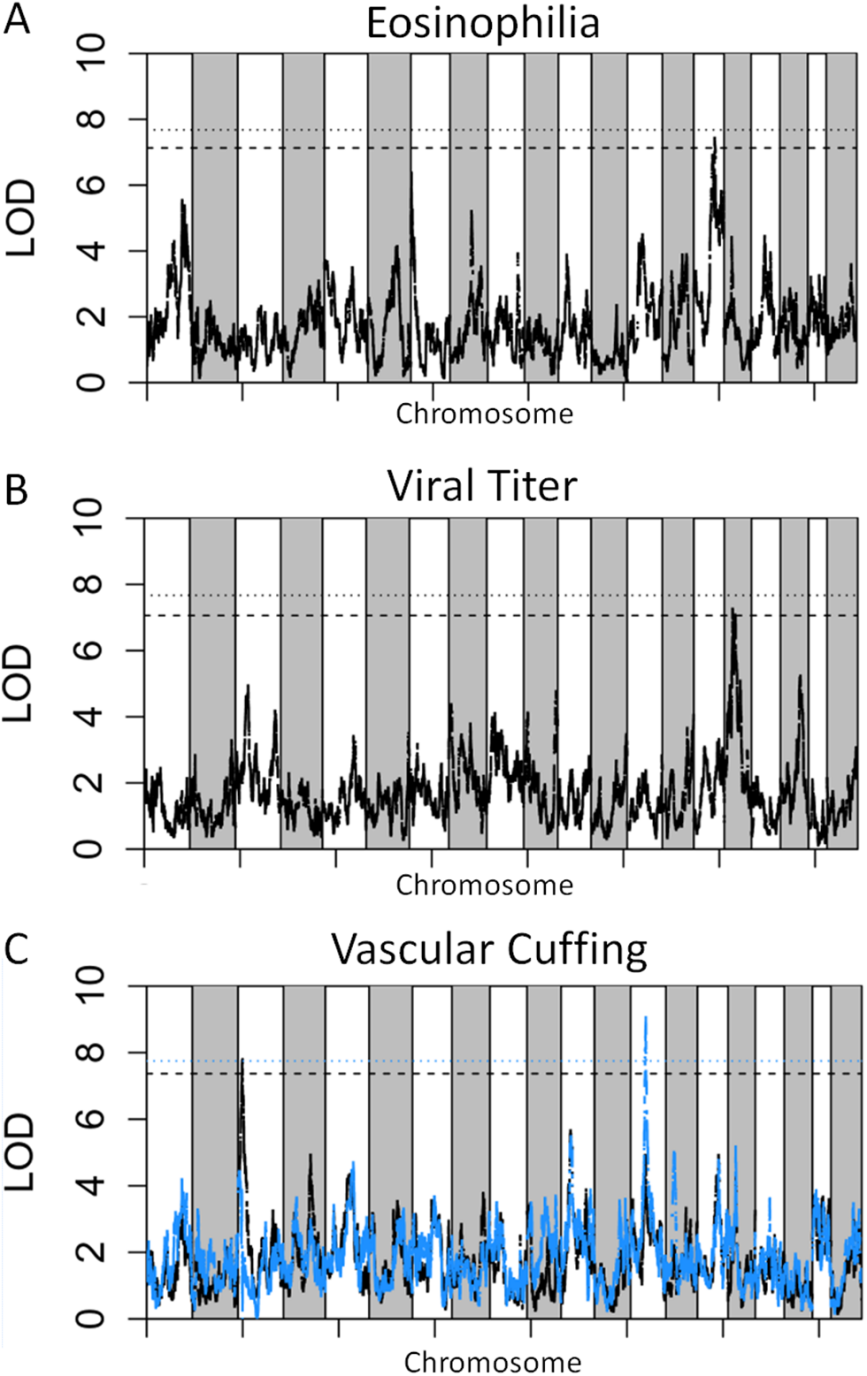
SARS QTL. Shown are LOD curves for each of three SARS-CoV associated phenotypes: (A) Eosinophilia, with *HrS3* on chromosome 15 responsible for 26% of phenotypic variation; (B) Viral titer with *HrS2* on chromosome 16 accounting for 22% of variation; and (C) Perivascular Cuffing, with *HrS1* (black curve) accounting for 26% of phenotypic variation, and *HrS4* (blue curve) accounting for 21% of phenotypic variation. In A and B, the upper horizontal dashed lines correspond to the genome-wide p = 0.05 significance threshold and the lower dashed lines correspond to a significance threshold of p = 0.1. In C the black dashed line corresponds to a genome-wide p = 0.05 significance threshold for *HrS1* and the blue dashed line corresponds to the genome-wide significance threshold of p = 0.05 for *Hrs4*, when conditioned on *HrS1*.

We genotyped 140 preCC animals at high density, including several that succumbed to infection prior to the scheduled day four harvest. As previously described [23,27], we conducted QTL mapping using Bagpipe (http://valdarlab.unc.edu/software.html) and the underlying eight founder strain haplotypes present in the CC to identify host genome regions containing polymorphisms significantly associated with SARS-induced disease responses. We identified four QTLs shown in **Fig 4**, *HrS1-4* (*H*ost *r*esponse to *S*ARS) that contributed to disease associated phenotypes at day four post infection. We identified a significant main effect QTL for vascular cuffing (Chr 3: 18286790–26668414), which explained 26% of the variation in vascular cuffing. We also identified two highly suggestive (genome-wide p-values based on permutations of 0.1>p>0.05) QTL for viral titer (Chr 16: 31583769–36719997) and eosinophil infiltration (Chr 15: 72103120–75803414), explaining 22% and 26% of the variation in these traits respectively. Finally, we also searched for modifier QTL, those QTL additively influencing a trait of interest, but whose presence was initially masked by our three other identified QTL. We identified a significant QTL further influencing vascular cuffing (Chr 13: 52822984–54946286), explaining an additional 21% of the variance in this phenotype. *HrS4* was a moderate peak even without considering *HrS1* status, suggesting that these interactions are additive. **Table 1** details each of the SARS susceptibility QTLs including LOD and p-values. Analysis of other phenotypes did not lead to discovery of QTLs passing the p<0.01 significance threshold.

**Table 1:** SARS QTL details. The four SARS-CoV susceptibility QTLs with genome region, LOD score, significance and percent of phenotypic variation.

QTL	Chromosome and Region	LOD	-logP	% variation	phenotype
**HrS1**	Chr 3: 18286790–26668414	7.79904	4.79028	26%	vascular cuffing
**HrS2**	Chr 16: 31583769–36719997	7.26072	4.37451	22%	titer
**HrS3**	Chr 15: 72103120–75803414	7.43370	4.49207	26%	eosinophilia
**HrS4**	Chr 13: 52822984–54946286	9.06019	5.34260	21%	vascular cuffing

### Integration of statistical, genetic and bioinformatic approaches identify high likelihood candidate genes

The genetic architecture of the preCC, with up to eight distinct haplotypes at each locus, provides unique opportunity for narrowing QTL regions to candidate genes or SNPs. To narrow QTL regions we estimated the additive allele effects associated with each haplotype and correlated these to the allelic states at candidate causative polymorphisms. Allele effects [23] describe the estimated effect of each of the eight founder haplotypes on the phenotype (e.g. a large positive allele effect for the PWK/PhJ haplotype suggests that having a PWK/PhJ allele will increase the phenotypic trait value of interest). In our analysis we focused on polymorphisms corresponding to the largest contrast between allele effects at the peak QTL locus. For *HrS1* we identified two haplotypes, C57BL/6J and WSB/EiJ increasing vascular cuffing relative to the haplotypes of the other six founder strains. For each of *HrS2-4*, we identified a single founder haplotype altering the phenotype relative to the seven other founder haplotypes (*HrS2*: PWK/PhJ haplotype reduced viral titer; *HrS3*: A/J haplotype increasing eosinophillic infiltration; *HrS4*: CAST/EiJ haplotype reduced vascular cuffing).

We then used high coverage whole genome sequence from the eight founder strains [37] to identify either private SNPs or small In/Dels in the case of a single causative haplotype, or regions of shared descent (in the case of two causative haplotypes) to narrow down the large QTL regions. *HrS1* was initially an 8.38 Mb region which contained 26 genes and 9 non-coding RNAs (ncRNAs). Identification of the sub-regions where C57BL/6J and WSB/EiJ share private, common ancestry reduced this region to 449 kb, which contained only one gene, one pseudogene and one miRNA of unknown function (*Trim55*, *GM7488* and AC107456.1, respectively). Allele effects for all four QTLs can be seen in **S3 Fig**.

The *HrS2* QTL on chromosome 16 was a 5.4 Mb region containing 92 genes and 30 ncRNAs. Across the eight founder strains, there were 95,936 SNPs or small In/Dels, and 33,288 of these were private to PWK/PhJ. Seven ncRNAs and 74 genes had private PWK/PhJ SNPs or In/Dels (**S3 Table**). We further prioritized these variants based on whether the PWK/PhJ private polymorphisms were likely to cause major functional changes to the gene (missense, nonsense, stop gained/lost, splice alterations or nonsense mediated decay). When we did so, we further reduced this list to 48 candidate genes including several mucins as well as genes involved in T cell activation and apoptosis.

The *HrS3* QTL on chromosome 15 was a 3.7 Mb region containing six ncRNAs and 63 genes. There were a total of 71,208 SNPs or small In/Dels in the region, 932 of which were private to A/J. No ncRNAs and only 25 genes contained a private A/J SNP or In/Del, and we further reduced these to one candidate gene with major functional changes (**S4 Table**), *Bai1*. *Bai1* is a high priority candidate gene given the association between eosinophils and angiogenesis [38]; however we have not chosen to focus on *Bai1* at this time because of the limited availability of tools for working on an A/J genetic background.

Finally, *HrS4* on chromosome 13 was a 2.12 Mb region containing three ncRNAs and 30 genes. There were a total of 461,46 SNPs or In/Dels in the region, 9,732 being private to CAST/EiJ. 29 of the genes and all three ncRNAs contained private CAST/EiJ polymorphisms (**S5 Table**). When we further prioritized based on major functional changes, we reduced the region to only one ncRNA and nine genes including *Cdhr2*, a member of the protocadherin family [39].

### *Trim55* deletion results in altered immune cell infiltration

**Fig 5:**
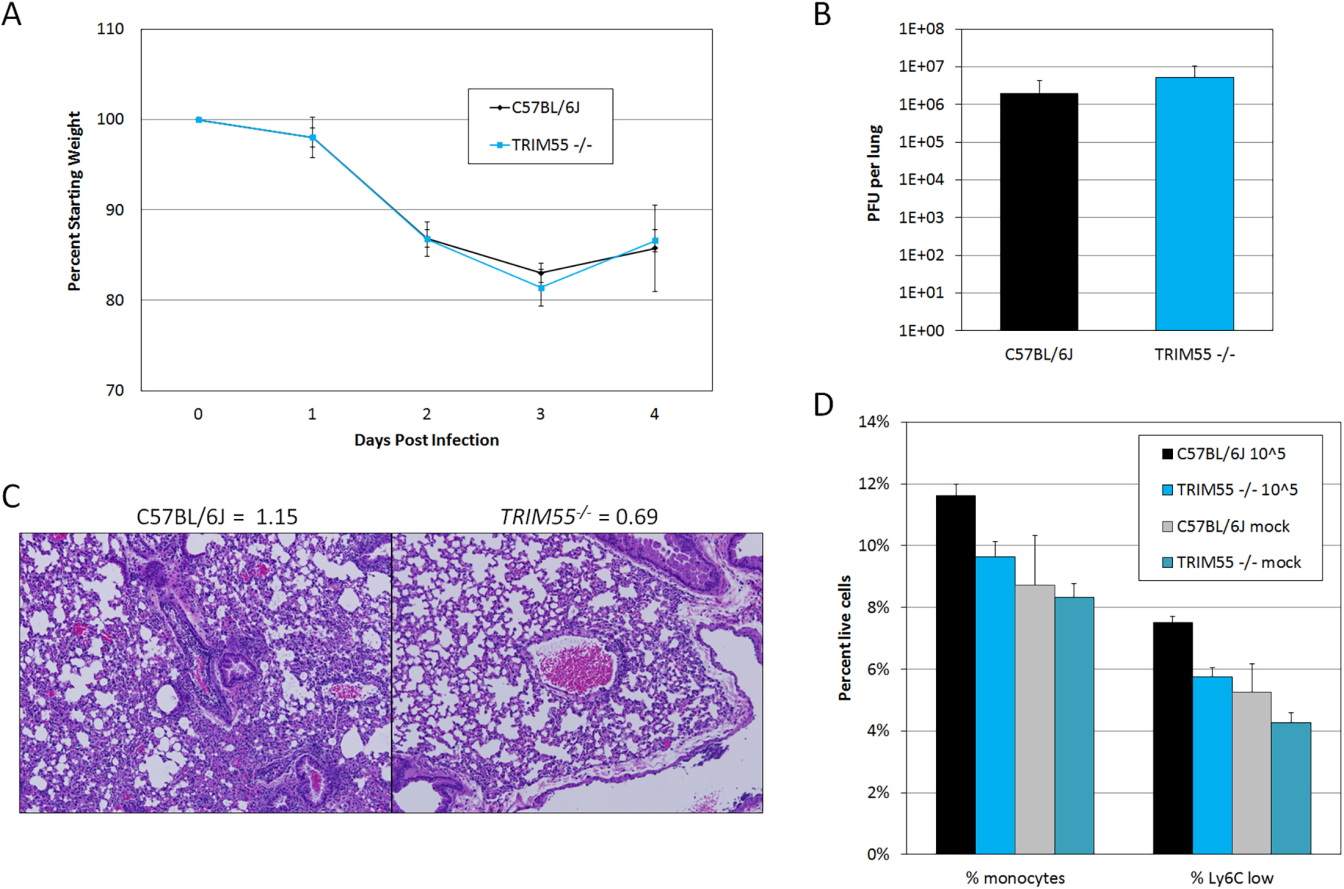
*Trim55* knockout phenotypes. (A) *Trim55* ^*-/-*^ and C57BL/6J control mice show similar weight loss over four days of infection. *Trim55* ^*-/-*^ are shown in gray, C57BL/6J controls are shown in black. (B) No differences were observed in lung titer at day four post infection. (C) Hematoxylin and eosin stained lung sections from *Trim55* ^*-/-*^ mice display less vascular cuffing than do those from C57BL/6J controls (p<0.05, t-test). (A-C). Three to ten *Trim55* ^*-/-*^ and C57BL/6J biological replicates were used, experiments were repeated at least three times. (D) SARS-CoV-infected *Trim55* ^*-/-*^ mice (n = 6) have lower percentages of monocytes (p<0.01, t-test), specifically Ly6C low staining monocytes (p = 0.001, t-test), in their lungs than do C57BL/6J controls (n = 6) as measured by flow cytometry. Mock infected mice have similar levels of monocytes and Ly6C low staining monocytes, respectively (p = 0.83 and p = 0.37, t-test). Experiment performed once. Data in A, B and D are means, error bars show standard error of the mean.

We focused our validation efforts on *Trim55*, the single *HrS1* candidate and a member of the TRIM protein superfamily which has not previously been associated with any infectious disease phenotype. Although many TRIM proteins function in innate immunity and inflammation, *Trim55* (also known as muscle-specific RING finger 2 or *Murf2*) has only been studied in the context of muscle development and cardiac function [40,41]. *Trim55* is expressed in smooth muscle surrounding blood vessels [42], an appropriate location to influence perivascular cuffing phenotypes. Knockout mice on a C57BL/6J background have previously been reported [43] and were kindly made available to our laboratory. Groups of age matched *Trim55* ^*-/-*^ and C57BL/6J control mice were infected with 10^5^ PFU of MA15 for four days and monitored daily for weight loss and signs of disease. *Trim55* ^*-/-*^ and C57BL/6J animals had similar weight loss profiles as well as similar viral loads in the lung at day four post infection (**Fig 5A and 5B**) and no differences in mortality. Hematoxylin and eosin stained lung sections showed significantly reduced vascular cuffing in the lungs of *Trim55* ^*-/-*^ (mean score of 0.69) compared to control animals (mean score of 1.15) (p < 0.05 by students t test, **Fig 5C**), confirming the role of *Trim55* in contributing to SARS-CoV-induced vascular cuffing. Additional mice were infected for flow cytometric analysis of inflammatory cell populations in the lung after MA15 infection. While we observed a general trend towards increased numbers of T cells, B cells and macrophages in the lungs of C57BL/6J control mice compared to the *Trim55* ^*-/-*^ mice, only monocyte numbers were significantly different between the two groups. Total monocytes, as well as the subset of Ly6C positive monocytes, were present in significantly higher numbers in the lungs of infected control mice compared to *Trim55* ^*-/-*^ mice (**Fig 5D**).

**Fig 6:**
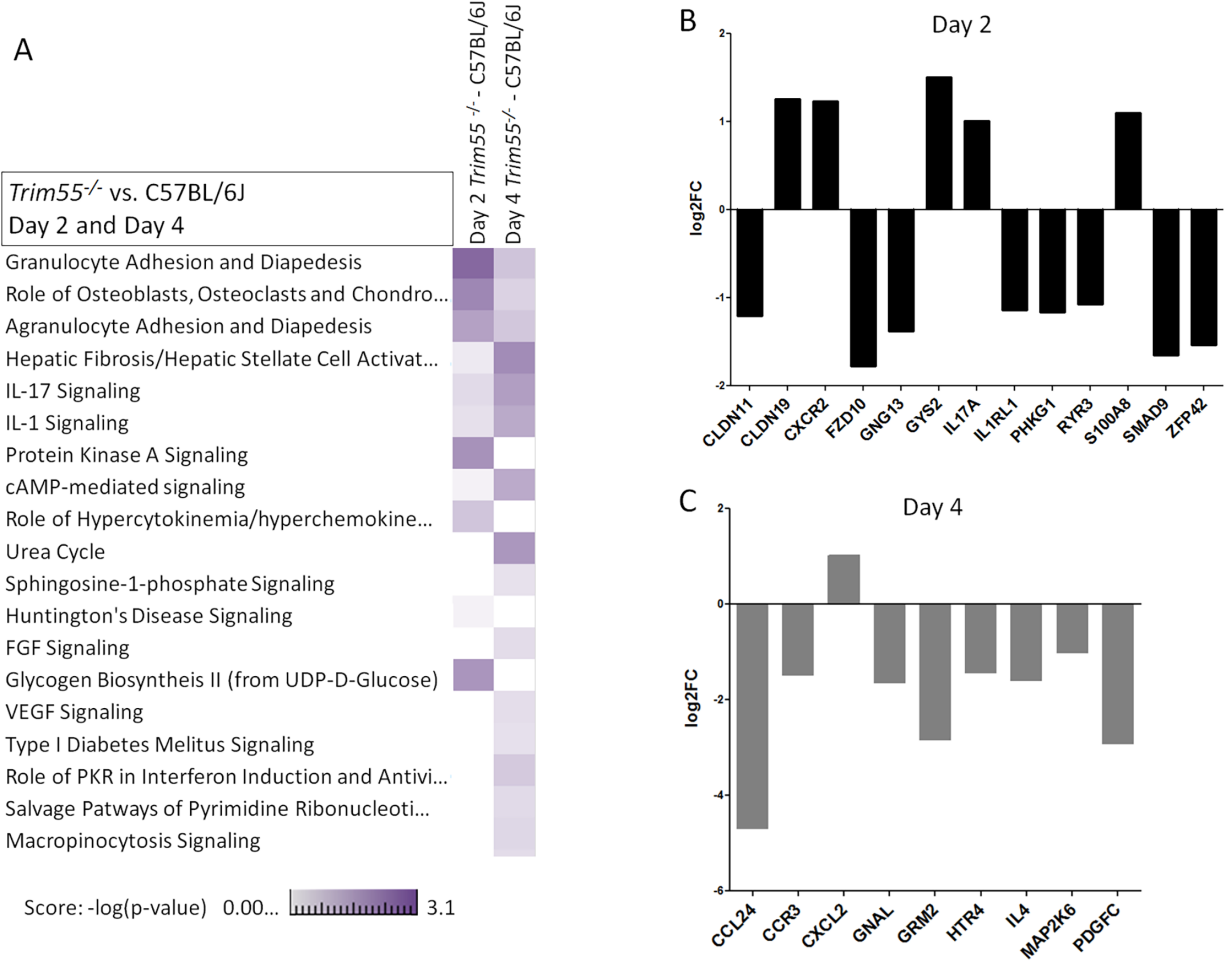
Transcriptional analysis. (A) A heat map showing a comparison of functionally enriched biological pathways between *Trim55* ^*-/-*^ and C57BL/6J mice at days two and four post infection based on RNA expression levels in the lung. Three mice used per condition, per timepoint. Experiment performed once. Relative expression of all DE genes (log2 FC of 1 or greater and FDR < .05, Trim55^-/-^ vs C57BL/6J) involved in granulocyte adhesion and diapedesis at day two (B) or four (C) post infection.

RNA was isolated from the lungs of mock and infected control and *Trim55* ^*-/-*^ mice at two and days four post infection. 168 genes were identified as differentially expressed (DE, log2 fold change >2 relative to mock) between the two strains, predominantly at day two post infection (GEO accession GSE64660). We then used Ingenuity Pathway Analysis software to identify functionally enriched gene categories. This analysis identified the granulocyte and agranulocyte diapedesis gene ontology categories as among the most significantly enriched (first and third respectively) from genes with DE between *Trim55* ^*-/-*^ and B6 controls (**Fig 6A**). Diapedesis, or extravasation, is the process by which inflammatory monocytes and leukocytes bind to endothelial cells and migrate from the blood stream into surrounding injured tissues. The transcriptional analysis indicates decreased expression of tight junction genes and increased chemokine expression in C57BL/6J mice compared to that observed in *Trim55* ^*-/-*^ mice. Relative expression of genes involved in granulocyte adhesion and diapedesis at days two and four post infection is shown in **Fig 6B and 6C**.

## Discussion

Emerging coronaviruses like SARS-CoV and MERS-CoV cause high morbidity and mortality in human populations. Because of limited access to clear human disease responses and samples from acute infections, as well as the limited number of overall infected individuals, it is extremely challenging to define the role of host genetic polymorphism in human disease. Coronavirus pathogenesis is heavily influenced by host genetics, as evidence by the extreme resistance of SJL mice, which encode a defective variant CEACAM1 receptor for mouse hepatitis virus entry and infection [44]. Furthermore, genetic monomorphisms in the cheetah have resulted in extreme hypersensitivity to feline infectious peritonitis coronavirus infection, underscoring the importance of abundant genetic variation in controlling lethal coronavirus infection [45,46]. In this study we examined numerous phenotypes following SARS-CoV infection and identified 4 QTL related to various aspects of SARS-CoV pathogenesis. These data support previous predictions that the CC platform can identify genetic variants contributing moderate effect sizes (e.g. ~20%) to complex immune response traits.

Two of the four identified QTL, on chromosome 3 and 13 respectively, pertained to perivascular cuffing. Perivascular cuffing in the lung is frequently observed during microbial and non-microbial lung disease [34,47,48] and is associated in part with extravasation, the process by which inflammatory cells migrate from the blood to surrounding tissues [49,50]. Previous reports of perivascular cuffing include lymphocyte and granulocyte involvement with limited insights into the genetic underpinnings of this phenotype. *In vivo* models of SARS-CoV infection have shown that vascular cuffing increases in cases of severe disease [21,22,35] and vascular congestion was observed in human SARS-CoV patients [7]. A recent study of pneumococcal infection [51] identified several QTL governing disease susceptibility including one on chromosome 13. The authors also found an association between perivascular inflammation and susceptibility to infection but did not extend their genetic analysis to that phenotype; there was no overlap between their chromosome 13 QTL and *Hrs4*. Analysis of pulmonary inflammation following hyperoxia-induced lung injury [52] identified QTL on chromosomes 1, 2, 4, 6 and 7 and informative SNPS helped to identify *Chrm2* as the causative gene on chromosome 6. In this study we identified QTL contributing to 26% and 21% of the total vascular cuffing phenotypic variance, respectively. The limited numbers of candidate genes under the larger effect size QTL allowed us to test and validate the role of *Trim55* in SARS-CoV-induced perivascular cuffing phenotype.

The CC was conceived to expand upon the genetic variation and mapping precision found within classical recombinant inbred (RI) panels which often suffer from inability to narrow the numbers of candidate genes due to the close genetic relationship of the founding lines. The classical BxD panel—derived from C57BL/6J and DBA2/J founder strains–was used previously to identify *Klra8*, the resistance gene to mouse cytomegalovirus (*Cmv–1*) infection [53]. Importantly, the validation experiments were conducted over a decade after the initial identification of the *Cmv–1* susceptibility locus [54] as the wide initial QTL interval was not sufficient for identification of specific candidate genes. The Collaborative Cross provides a significant advantage in comparison to two-way crosses and other bi-allelic RI strain panels–as illustrated by our study, allele effects associated with founder haplotypes can provide a substantial reduction in the list of plausible candidate loci. Moreover, the inclusion of a diverse set of founder strains increases the likelihood of variants existing at loci that can influence any given trait. Indeed, in our study five of the eight founder strains contributed minor, causative alleles to the four QTL we identified. As the breeding of the CC lines preserved genetic variation across the genome, the CC lacks genetic blind spots and has multiple variant alleles at each locus. With a wide range of phenotypes [23], the CC recapitulates aspects of the genetic diversity of the human population, making it a powerful system for use in causal genetic analyses.

This study was part of an early pilot project to demonstrate the utility of the CC panel [23]. As such we did not have access to fully inbred animals and were limited to a single animal per genotype. However, the increased control of the experimental conditions in these studies and high frequency of minor alleles within the CC population (each allele is present in roughly 12.5% of CC genomes [23] whereas minor allele frequencies in the human population are typically much lower) allowed us to identify multiple host genome regions contributing to differential SARS-CoV infection. Studies utilizing the full CC panel will be able to use the full potential of a reproducible genetic background to obtain repeated assays and high-precision phenotyping, even our limited proof-of-concept study proved to be adequate to identify multiple host genome regions contributing to differential responses to SARS-CoV infection.

*Trim55* is part of the well-known superfamily of TRIM proteins, specifically in the C-II subfamily. This subfamily consists of *Trim54*, *Trim55* and *Trim63*, and is defined by an N-terminal that contains a Ring Finger domain, B-box 2 domain and a coiled-coil domain [42]. The C-II Trim family genes are solely expressed by muscle cells and to date have only been studied in the frame of muscle cell development and cardiac function. *Trim55* and *Trim63*, also known as *Murf1*, mediate muscle cell protein turnover through their E3 ubiquitin-ligase activities and function in muscle wasting phenotypes [40,43,55]. *Trim55* specifically functions in myosin and myofibril maintenance and knockdown studies correlate *Trim55* levels with modified post-translational microtubule modifications and defects in myofibril assembly, critical components in extravasation [55].

Blood vessels are comprised of vascular endothelial cells, connective tissue and smooth muscle cells, all of which must be crossed during inflammatory cell trafficking into the lung. During extravasation, inflammatory cells tumble and bind to adhesion molecules, slowing their motion and expanding surface-surface interactions with endothelial cells [56]. *Tissue Necrosis Factor-alpha* and *thrombin* expression levels increase following SARS-CoV infection [12,21] and these proteins have both been shown to increase endothelial permeability [57,58]. Here we observed a complicated picture of altered chemokine and tight junction gene expression in the absence of *Trim55* (**Fig 6B and 6C**). Increased expression of *Ccl24*, *CCR3*, *IL4* and *Pdgfc* in C57BL/6J mice compared to that in *Trim55* ^*-/-*^ mice at day four post infection correlates with increased inflammatory cell recruitment and binding to extracellular matrix proteins. These expression changes are consistent with changes in altered recruitment of inflammatory cells to the lung following SARS-CoV infection. Higher expression of *Claudin19* at day two post infection in *Trim55* deficient mice likely contributes to decreased tight junction permeability and reduced ability for inflammatory cells in the bloodstream to cross the endothelial barrier. Additionally, one of the high priority candidate genes under the modifier QTL on chromosome 13 is *Cdhr2*, a cadherin superfamily member that may also play a role in extravasation of inflammatory cells into the infected lung. Different specific VE-cadherin residues are known to regulate leukocyte extravasation and vascular permeability [59], demonstrating the importance of cadherin family members in these processes. More recent work details the role of *Cdhr2* in intestinal brush border assembly via adhesion links between adjacent microvilli [60].

Intravascular crawling and signaling through RhoA induces actin, microfilament and microtubule reorganizations and the production of endothelial cell docking structures, which surround the inflammatory cell and span tight junctions [56]. Although controversial, myofibril contractile structures may also contribute in to the assembly of these structures. In any event, inflammatory cell transmigration requires the formation of actin-myosin II contractile structures which are attached to tight junction membranes by VE-cadherins, resulting in increased endothelial tension, and programmed separation and expansion of the tight junctions which allow for leukocyte/monocyte passage into the surrounding tissues [61]. It seems likely that *Trim55*, with its roles in myosin and myofibril maintenance and microtubule organization, contributes to the programmed formation of endothelial docking structures and regulation of inflammatory cell transmigration; key features associated with the formation of perivascular cuffs around vessels in the lung. Our data (**Figs 5 and 6**) demonstrate that *Trim55* contributes to vascular cuffing following SARS-CoV infection. While the mechanism is not yet fully understood, the data strongly suggest that *Trim55* is important for extravasation of inflammatory cells, and thus overall SARS-CoV pathogenesis, by altering intercellular junctions and chemotactic signals. Increased studies of *Trim55* and *Cdhr2* function within the CC population, either via specific crosses of lines with high and low alleles at the *HrS1* and *HrS4* loci, or via CRISPR-Cas9 modification of these loci will allow further insight into the role that these two genes play during SARS-CoV pathogenesis and recovery, as well as increasing understanding of the more general process of extravasation.

The Collaborative Cross was conceived of as a resource to drive insight into a variety of biomedically important diseases via the reassortment of genetic variants and expansion of phenotypic ranges [62]. Indeed, previous studies with various preCC subsets have demonstrated expanded phenotypes in preCC mice body weight and hematological parameters [23,63,64], response to Aspergillus [65] and susceptibility to Influenza A infection [27,66]. More recently it has been shown that novel combinations of alleles have also resulted in new models for human disease such a spontaneous colitis [67], and that F1 hybrids of CC mice were used to create an improved mouse model for Ebola virus disease [68] including hemorrhagic signs of disease previously not observed in a small animal model. Within our study of SARS-CoV infection within the preCC, we showed more extreme disease phenotypes than those seen within the eight founder strains of the CC. These disease phenotypes included virus titer, weight loss, pathology and lethality. Further, we saw the emergence of new phenotypes including ARDS and DAD not traditionally seen within young inbred strains [11]. Importantly, our results highlight another exciting aspect of the nature of CC genome: transgressive segregation, or the release of cryptic genetic variation [69,70]. As the three wild-derived CC founders all showed mortality early in the course of SARS-CoV infection, genetic variants within these three strains impacting later-stage SARS-CoV responses would normally not be seen. Only via the reassortment of these alleles into a variety of genetic backgrounds (some resistant to clinical disease, some susceptible) were we able to show that alleles from all three wild-derived founders impacted perivascular cuffing or viral titer levels independent of their effects on clinical disease or SARS-CoV mortality. Collectively, these data support the hypothesis that the CC population represents a robust platform for developing improved animal models that more readily replicate disease phenotypes seen in human populations. All told, our identification of multiple QTL related to SARS-CoV pathogenesis, identification of a novel function for *Trim55*, and the development of new models of acute lung injury, further solidify the utility of the CC as a valuable community resource for research of infectious diseases and other biological systems driven by complex host response networks.

## Materials and Methods

### Ethics statement

Mouse studies were performed in strict accordance with the recommendations in the Guide for the Care and Use of Laboratory Animals of the National Institutes of Health. All mouse studies were performed at the University of North Carolina (Animal Welfare Assurance #A3410-01) using protocols approved by the UNC Institutional Animal Care and Use Committee (IACUC).

### Virus and cells

Recombinant mouse-adapted SARS-CoV (MA15) was propagated and titered on Vero E6 cells. For virus titration half of the right lung was used to assess plaque forming units (PFU) per lung using Vero E6 cells with a detection limit of 100 PFU [71]. All experiments were performed in a Class II biological safety cabinet in a certified biosafety level 3 laboratory containing redundant exhaust fans while wearing personnel protective equipment including Tyvek suits, hoods, and HEPA-filtered powered air-purifying respirators (PAPRs).

### Animals

8–12 week old female animals from the 8 founder strains (A/J, C57BL/6J, 129S1/SvImJ, NOD/ShiLtJ, NZO/HILtJ, CAST/EiJ, PWK/PhJ, and WSB/EiJ) were obtained from the Jackson labs (jax.org), and bred at UNC Chapel Hill under specific pathogen free conditions. 8–20 week old female pre-CC mice were bred at Oak Ridge National Laboratories under specific pathogen free conditions, and transferred directly into a BSL–3 containment laboratory at UNC Chapel Hill. One preCC mouse per line was infected, for the founder strains at day four n = 2 (A/J), n = 3 (C57BL/6J, 128S1/SvImJ, NOD/ShiLtJ, CAST/EiJ, PWK/PhJ and WSB/EiJ) and n = 5 (NZO/HILtJ). *Trim55* ^-/-^ (*Murf2* ^-/-^) mice on a C57BL/6 background were a kind gift from Christian Witt at the University of Mannheim. Validation experiments used 8–12 week old female mice. All experiments were approved by the UNC Chapel Hill Institutional Animal Care and Use Committee. Animals were maintained in SealSafe ventilated caging system in a BSL3 laboratory, equipped with redundant fans as previously described by our group.

### Infections

Animals were lightly anesthetized via inhalation with Isoflurane (Piramal). Following anesthesia, animals were infected intranasally with 10^5^ pfu of mouse adapted SARS-CoV (MA15) in 50 μL of phosphate buffered saline (PBS, Gibco), while mock infected animals received only 50 μL of PBS. Animals were weighed daily and at four days post infection, animals were euthanized via Isoflurane overdose and tissues were taken for various assays. No blinding was used in any animal experiments and animals were not randomized; group sample size was chosen based on availability of age-matched mice. Pearson’s correlation was used to determine any correlation between weight loss and log-transformed viral load in the lung.

### Histological analysis

The left lung was removed and submerged in 10% buffered formalin (Fisher) without inflation for 1 week. Tissues were embedded in paraffin, and 5 μm sections were prepared by the UNC Lineberger Comprehensive Cancer Center histopathology core facility. To determine the extent of inflammation, sections were stained with hematoxylin and eosin (H & E) and scored in a blinded manner by for a variety of metrics relating to the extent and severity of immune cell infiltration and pathological damage on a 0–3 (none, mild, moderate, severe) scale. Significant differences in lung pathology were determined by a two-sample student’s t test. Images were captured using an Olympus BX41 microscope with an Olympus DP71 camera.

### Flow cytometry

The right lung of each mouse was used for flow cytometric staining of inflammatory cells. Mice were perfused with PBS through the right ventricle before harvest, lung tissue was dissected and digested in RPMI (Gibco) supplemented with DNAse and Collagenase (Roche). Samples were strained using a 70 micron filter (BD) and any residual red blood cells were lysed using ACK lysis buffer. The resulting single cell suspension was stained with two antibody panels using the following stains (1) FITC anti-Ly-6C clone AL21 (BD), PE anti-SigLecF clone E50-2440 (BD), PETR anti-CD11c clone N418 (MP), PerCP anti-B220 clone RA3-6B2 (MP), PE-Cy7 anti-Gr–1 clone RB6-8C5 (eBio), eF450 anti-CD11b clone M1/70 (eBio), APC anti-LCA clone 30-F11 (eBio), APC-eF780 anti-MHC class II clone M5/114 (eBio) or (2) FITC anti-CD94 clone 18d3 (eBio), PE anti-CD3Ɛ clone 145-2C11 (eBio), PETR anti-CD4 clone RM4-5 (MP), PerCP anti-CD8 clone 53–6.7 (BD), PE-Cy7 anti-CD49b clone DX5 (eBio), eF450 anti-LCA clone 30-F11 (eBio), AF647 anti-CD19 clone 6D5 (Biolegend), APC-eF780 anti-B220 clone RA3-6B2 (eBio). While this FACS analysis was solely performed on mice of a C57BL6/J background, these antibodies have all been shown recognize the relevant antigens in each of the CC founder lines. Samples were run on a Beckman Coulter CyAN, and data analyzed within the Summit software. Significant differences in lung inflammatory cell populations were determined by a two-sample student’s t test.

### Genotyping and haplotype reconstruction

Genotyping and haplotype reconstruction were done as described in [23]. Briefly, each pre-CC animal was genotyped using the Mouse Diversity Array [72] (Affymetrix) at 372,249 well performing SNPs which were polymorphic across the founder strains [31]. Once genotypes were determined, founder strain haplotype probabilities were computed for all genotyped loci using the HAPPY algorithm [73]. Genetic map positions were based on the integrated mouse genetic map using mouse genome build 37 [74].

### Linkage mapping and identification of candidate regions

Linkage mapping was done as described in [23]. Briefly, QTL mapping was conducted using the BAGPIPE package [75] to regress each phenotype on the computed haplotypes in the interval between adjacent genotype markers, producing a LOD score in each interval to evaluate significance. Genome-wide significance was determined by permutation test, with 250 permutations conducted per scan. Phenotype data for mapping either satisfied the assumptions of normality or were log transformed to fit normality (titer data). For the likely regions of identified QTL peaks, SNP data for the eight founder strains from the Sanger Institute Mouse Genomes Project [37] were downloaded and analyzed as described in Ferris et al [27].

### Code availability

Bagpipe is freely available at http://valdarlab.unc.edu/software.html.

### RNA preparation and oligonucleotide microarray processing

At two and four days after infection, mice were euthanized and a lung portion placed in RNAlater (Applied Biosystems/Ambion) and then stored at −80°. The tissues were subsequently homogenized in TriZol (Life Technologies), and RNA extracted as previously described [12]. RNA samples were spectroscopically verified for purity, and the quality of the intact RNA was assessed using an Agilent 2100 Bioanalyzer. cRNA probes were generated from each sample by the use of an Agilent one-color Quick-Amp labeling kit. Each cRNA sample was then hybridized to Agilent mouse whole-genome oligonucleotide microarrays (4 x 44) based on the manufacturer’s instructions. Slides were scanned with an Agilent DNA microarray scanner, and the output images were then analyzed using Agilent Feature Extractor software. Microarray data has been deposited in the National Center for Biotechnology Information’s Gene Expression Omnibus database and is accessible through GEO accession GSE64660.

### Microarray data analysis/methods

Raw Agilent Microarray files were feature extracted Agilent feature extractor version 10.7.3.1. Raw Microarray files were background corrected using the “norm-exp” method with an offset of 1 and quantile normalized using Agi4x44PreProcess [76] in the R statistical software environment. Replicate probes were mean summarized, and all probes were required to pass Agilent QC flags for 75% replicates of at least one infected time point (41,267 probes passed). This microarray analysis was performed only on animals with a C56BL6/J background; thus it was not necessary to correct for probes with SNPs caused by the genetic variation of the other founder lines.

### Statistical analysis

Differential expression was determined by comparing MA15 infected samples (C57Bl6/J mice vs. *Trim55* ^*-/-*^ mice) with mock and each other to fit a linear model for each probe using the R package Limma. Criteria for differential expression was an absolute log2 FC of 1 and a q value of < 0.05 calculated using a moderated t test with subsequent Benjamini-Hochberg correction. Differentially expressed (DE) genes were observed for both C57BL/6J and *Trim55* ^*-/-*^ infected mice compared to time matched mocks at two and day four post infection. DE analysis was also run on the *Trim55* ^*-/-*^ infected mice against the C57BL/6J infected mice which provided a direct observation of the transcription signatures in the *Trim55* ^*-/-*^ against the MA15 infected mouse background. To identify genes with similar patterns of variation at early and late times post infection, day two and day four gene signatures were intersected separately and then combined. There was no intersection of DE genes between the day two and four time points when the *Trim55* ^*-/-*^ infected mice were run against the C57BL/6J infected mice.

### Functional analysis

Functional analysis of statistically significant gene expression changes was performed using the Ingenuity Pathways Knowledge Base (IPA; Ingenuity Systems) [77]. Functional enrichment scores were calculated in IPA using all probes that passed our QC filter as the background data set.

## Supporting Information

S1 Fig Founder susceptibility to SARS-CoV. Weight loss (A), survival (B) and viral load (C) in the lung following infection with 10^5^ PFU of SARS-CoV.
(TIF)

S2 Fig Pathology quantitation. (A) Total pathology scores for each preCC line were summed and then grouped. (B) Breakdown of preCC lines by eosinophilia score. (C) Breakdown of preCC lines by airway denudation score. (D) Breakdown of preCC lines by vascular cuffing score.
(TIF)

S3 Fig Allele effects. Effects of the founder alleles are shown for *HrS1*, *HrS2*, *HrS3 and HrS4*. The y-axis indicates the effect of a single founder allele in the QTL region on the overall phenotype.
(TIF)

S1 Table Wild-derived strain susceptibility. LD50 was determined for each of the wild-derived founder strains used in the CC.
(DOCX)

S2 Table Full pathology data. Full pathology scoring for each preCC line broken down by airway disease, vasculature, alveoli/parenchyma, DAD and presence of eosinophils.
(XLSX)

S3 Table *Hrs2* candidates. (DOCX)

S4 Table *Hrs3* candidates. (DOCX)

S5 Table *Hrs4* candidates. (DOCX)

S1 Dataset PreCC and founder phenotypes. (XLSX)

S2 Dataset *Trim55* ^*-/-*^ phenotypes. (XLSX)

## References

[1] Peiris JS, Lai ST, Poon LL, Guan Y, Yam LY, et al (2003) Coronavirus as a possible cause of severe acute respiratory syndrome. Lancet 361: 1319–1325. 12711465 10.1016/S0140-6736(03)13077-2 PMC7112372

[2] Lau SK, Woo PC, Li KS, Huang Y, Tsoi HW, et al (2005) Severe acute respiratory syndrome coronavirus-like virus in Chinese horseshoe bats. Proc Natl Acad Sci U S A 102: 14040–14045. 16169905 10.1073/pnas.0506735102 PMC1236580

[3] Peiris JS, Chu CM, Cheng VC, Chan KS, Hung IF, et al (2003) Clinical progression and viral load in a community outbreak of coronavirus-associated SARS pneumonia: a prospective study. Lancet 361: 1767–1772. 12781535 10.1016/S0140-6736(03)13412-5 PMC7112410

[4] Chan JW, Ng CK, Chan YH, Mok TY, Lee S, et al (2003) Short term outcome and risk factors for adverse clinical outcomes in adults with severe acute respiratory syndrome (SARS). Thorax 58: 686–689. 12885985 10.1136/thorax.58.8.686 PMC1746764

[5] Booth CM, Matukas LM, Tomlinson GA, Rachlis AR, Rose DB, et al (2003) Clinical features and short-term outcomes of 144 patients with SARS in the greater Toronto area. JAMA 289: 2801–2809. 12734147 10.1001/jama.289.21.JOC30885

[6] He Z, Zhao C, Dong Q, Zhuang H, Song S, et al (2005) Effects of severe acute respiratory syndrome (SARS) coronavirus infection on peripheral blood lymphocytes and their subsets. Int J Infect Dis 9: 323–330. 16095942 10.1016/j.ijid.2004.07.014 PMC7110876

[7] Franks T, Chong P, Chui P, Galvin J, Lourens R, et al (2003) Lung pathology of severe acute respiratory syndrome (SARS): a study of 8 autopsy cases from Singapore. Human Pathology 34: 743–748. 14506633 10.1016/S0046-8177(03)00367-8 PMC7119137

[8] Ip WK, Chan KH, Law HK, Tso GH, Kong EK, et al (2005) Mannose-binding lectin in severe acute respiratory syndrome coronavirus infection. J Infect Dis 191: 1697–1704. 15838797 10.1086/429631 PMC7199483

[9] Wang Y, Yan J, Shi Y, Li P, Liu C, et al (2009) Lack of association between polymorphisms of MASP2 and susceptibility to SARS coronavirus infection. BMC Infect Dis 9: 51 10.1186/1471-2334-9-51 19405982 PMC2683852

[10] Roberts A, Deming D, Paddock C, Cheng A, Yount B, et al (2007) A mouse adapted SARS coronavirus causes disease and mortality in BALB/c mice. Plos Pathog 3(1): e5 17222058 10.1371/journal.ppat.0030005 PMC1769406

[11] Rockx B, Sheahan T, Donaldson E, Harkema J, Sims AC, et al (2007) Synthetic Reconstruction of Zoonotic and Early Human SARS-CoV Isolates that Produce Fatal Disease in Aged Mice. J Virol May 16: epub. 10.1128/JVI.00505-07 PMC1933338 17507479

[12] Gralinski LE, Bankhead A 3rd, Jeng S, Menachery VD, Proll S, et al (2013) Mechanisms of severe acute respiratory syndrome coronavirus-induced acute lung injury. MBio 4: pii: e00271-00213. 10.1128/mBio.00271-13 23919993 PMC3747576

[13] Zaki AM, van Boheemen S, Bestebroer TM, Osterhaus AD, Fouchier RA (2012) Isolation of a novel coronavirus from a man with pneumonia in Saudi Arabia. N Engl J Med 367: 1814–1820. 10.1056/NEJMoa1211721 23075143

[14] Raj VS, Farag EA, Reusken CB, Lamers MM, Pas SD, et al (2014) Isolation of MERS Coronavirus from a Dromedary Camel, Qatar, 2014. Emerg Infect Dis 20: 1339–1342. 10.3201/eid2008.140663 25075761 PMC4111206

[15] Corman VM, Ithete NL, Richards LR, Schoeman MC, Preiser W, et al (2014) Rooting the phylogenetic tree of MERS-Coronavirus by characterization of a conspecific virus from an African Bat. J Virol 88: 11297–11303. 10.1128/JVI.01498-14 25031349 PMC4178802

[16] Dragic T, Litwin V, Allaway GP, Martin SR, Huang Y, et al (1996) HIV–1 entry into CD4+ cells is mediated by the chemokine receptor CC-CKR–5. Nature 381: 667–673. 8649512 10.1038/381667a0

[17] Lindesmith L, Moe C, Marionneau S, Ruvoen N, Jiang X, et al (2003) Human susceptibility and resistance to Norwalk virus infection. Nature medicine 9: 548–553. 12692541 10.1038/nm860

[18] Ge D, Fellay J, Thompson AJ, Simon JS, Shianna KV, et al (2009) Genetic variation in IL28B predicts hepatitis C treatment-induced viral clearance. Nature 461: 399–401. 10.1038/nature08309 19684573

[19] Zhang H, Zhou G, Zhi L, Yang H, Zhai Y, et al (2005) Association between mannose-binding lectin gene polymorphisms and susceptibility to severe acute respiratory coronavirus infection. J Infectious Dis 192: 1355–1361. 16170752 10.1086/491479 PMC7202438

[20] Yuan FF, Tanner J, Chan PK, Biffin S, Dyer WB, et al (2005) Influence of FcgammaRIIA and MBL polymorphisms on severe acute respiratory syndrome. Tissue Antigens 192: 1355–1361. 10.1111/j.1399-0039.2005.00476.x PMC7190181 16185324

[21] Sheahan T, Morrison TE, Funkhouser W, Uematsu S, Akira S, et al (2008) MyD88 is required for protection from lethal infection with a mouse-adapted SARS-CoV. Plos Pathog 4: e1000240 10.1371/journal.ppat.1000240 19079579 PMC2587915

[22] Frieman MB, Chen J, Morrison TE, Whitmore AC, Funkhouser W, et al (2010) SARS-CoV pathogenesis is regulated by a STAT1 dependent but a type I, II and III interferon receptor independent mechanism. Plos Pathog 6: e1000849 10.1371/journal.ppat.1000849 20386712 PMC2851658

[23] Aylor DL, Valdar W., Foulds-Mathes W., Buus R.J., Verdugo R.A., Baric R.S., Ferris M.T., Frelinger J.A., Heise M., Frieman M.B., et al (2011) Genetic analysis of complex traits in the emerging Collaborative Cross. Genome Res 21: 1213–1222. 10.1101/gr.111310.110 21406540 PMC3149489

[24] Churchill GA, Airey DC, Allayee H, Angel JM, Attie AD, et al (2004) The Collaborative Cross, a community resource for the genetic analysis of complex traits. Nat Genet 36: 1133–1137. 15514660 10.1038/ng1104-1133

[25] Consortium CC (2012) The genome architecture of the Collaborative Cross mouse genetic reference population. Genetics 190: 389–401. 10.1534/genetics.111.132639 22345608 PMC3276630

[26] Threadgill DW, Miller DR, Churchill GA, de Villena FP (2011) The collaborative cross: a recombinant inbred mouse population for the systems genetic era. ILAR J 52: 24–31. 21411855 10.1093/ilar.52.1.24

[27] Ferris MT, Aylor DL, Bottomly D, Whitmore AC, Aicher LD, et al (2013) Modeling host genetic regulation of influenza pathogenesis in the collaborative cross. PLoS Pathog 9: e1003196 10.1371/journal.ppat.1003196 23468633 PMC3585141

[28] Kadarmideen HN, von Rohr P, Janss LL (2006) From genetical genomics to systems genetics: potential applications in quantitative genomics and animal breeding. Mamm Genome 17: 548–564. 16783637 10.1007/s00335-005-0169-x PMC3906707

[29] Flint J, Valdar W, Shifman S, Mott R (2005) Strategies for mapping and cloning quantitative trait genes in rodents. Nat Rev Genet 6: 271–286. 15803197 10.1038/nrg1576

[30] Valdar W, Flint J, Mott R (2006) Simulating the collaborative cross: power of quantitative trait loci detection and mapping resolution in large sets of recombinant inbred strains of mice. Genetics 172: 1783–1797. 16361245 10.1534/genetics.104.039313 PMC1456308

[31] Yang H, Wang JR, Didion JP, Buus RJ, Bell TA, et al (2011) Subspecific origin and haplotype diversity in the laboratory mouse. Nat Genet 43: 648–655. 10.1038/ng.847 21623374 PMC3125408

[32] Roberts A, Pardo-Manuel de Villena F, Wang W, McMillan L, Threadgill DW (2007) The polymorphism architecture of mouse genetic resources elucidated using genome wide resequencing data: implications for QTL discovery and systems genetics. Mamm Genome 18: 473–481. 17674098 10.1007/s00335-007-9045-1 PMC1998888

[33] Kwon D, Shin K, Kim S, Ha Y, Choi JH, et al (2010) Replication and pathogenesis of the pandemic (H1N1) 2009 influenza virus in mammalian models. J Microbiol 48: 657–662. 10.1007/s12275-010-0120-z 21046344

[34] Shubitz LF, Dial SM, Perrill R, Casement R, Galgiani JN (2008) Vaccine-induced cellular immune responses differ from innate responses in susceptible and resistant strains of mice infected with Coccidioides posadasii. Infect Immun 76: 5553–5564. 10.1128/IAI.00885-08 18852250 PMC2583549

[35] Clay CC, Donart N, Fomukong N, Knight JB, Overheim K, et al (2014) Severe acute respiratory syndrome-coronavirus infection in aged nonhuman primates is associated with modulated pulmonary and systemic immune responses. Immun Ageing 11: 4 10.1186/1742-4933-11-4 24642138 PMC3999990

[36] Lowe K, Alvarez DF, King JA, Stevens T (2010) Perivascular fluid cuffs decrease lung compliance by increasing tissue resistance. Crit Care Med 38: 1458–1466. 10.1097/CCM.0b013e3181de18f0 20400904 PMC2908493

[37] Keane TM, Goodstadt L, Danecek P, White MA, Wong K, et al (2011) Mouse genomic variation and its effect on phenotypes and gene regulation. Nature 477: 289–294. 10.1038/nature10413 21921910 PMC3276836

[38] Loffredo S, Staiano RI, Granata F, Genovese A, Marone G (2014) Immune cells as a source and target of angiogenic and lymphangiogenic factors. Chem Immunol Allergy 99: 15–36. 10.1159/000353316 24217601

[39] Ose R, Yanagawa T, Ikeda S, Ohara O, Koga H (2009) PCDH24-induced contact inhibition involves downregulation of beta-catenin signaling. Mol Oncol 3: 54–66. 10.1016/j.molonc.2008.10.005 19383367 PMC5527873

[40] Pizon V, Iakovenko A, Van Der Ven PF, Kelly R, Fatu C, et al (2002) Transient association of titin and myosin with microtubules in nascent myofibrils directed by the MURF2 RING-finger protein. J Cell Sci 115: 4469–4482. 12414993 10.1242/jcs.00131

[41] Willis MS, Wadosky KM, Rodriguez JE, Schisler JC, Lockyer P, et al (2014) Muscle ring finger 1 and muscle ring finger 2 are necessary but functionally redundant during developmental cardiac growth and regulate E2F1-mediated gene expression in vivo. Cell Biochem Funct 32: 39–50. 10.1002/cbf.2969 23512667 PMC3728166

[42] Ozato K, Shin DM, Chang TH, Morse HC 3rd (2008) TRIM family proteins and their emerging roles in innate immunity. Nat Rev Immunol 8: 849–860. 10.1038/nri2413 18836477 PMC3433745

[43] Witt CC, Witt SH, Lerche S, Labeit D, Back W, et al (2008) Cooperative control of striated muscle mass and metabolism by MuRF1 and MuRF2. EMBO J 27: 350–360. 18157088 10.1038/sj.emboj.7601952 PMC2168395

[44] Hirai A, Ohtsuka N, Ikeda T, Taniguchi R, Blau D, et al Role of mouse hepatitis virus (MHV) receptor murine CEACAM1 in the resistance of mice to MHV infection: studies of mice with chimeric mCEACAM1a and mCEACAM1b. J Virol 84: 6654–6666. 10.1128/JVI.02680-09 20410265 PMC2903249

[45] Evermann JF, Heeney JL, Roelke ME, McKeirman AJ, O'Brien SJ (1988) Biological and pathological consequences of feline infectious peritonitis virus infection in the cheetah. Arch Virol 102: 155–171. 2849387 10.1007/BF01310822 PMC7087010

[46] O'Brien SJ, Roelke ME, Marker L, Newman A, Winkler CA, et al (1985) Genetic basis for species vulnerability in the cheetah. Science 22: 1428–1434. 10.1126/science.2983425 2983425

[47] Sureshkumar V, Paul B, Uthirappan M, Pandey R, Sahu AP, et al (2005) Proinflammatory and anti-inflammatory cytokine balance in gasoline exhaust induced pulmonary injury in mice. Inhal Toxicol 17: 161–168. 15788377 10.1080/08958370590904616

[48] Mehlhop PD, van de Rijn M, Goldberg AB, Brewer JP, Kurup VP, et al (1997) Allergen-induced bronchial hyperreactivity and eosinophilic inflammation occur in the absence of IgE in a mouse model of asthma. Proc Natl Acad Sci U S A 94: 1344–1349. 9037055 10.1073/pnas.94.4.1344 PMC19793

[49] Goldblum SE, Hennig B, Jay M, Yoneda K, McClain CJ (1989) Tumor necrosis factor alpha-induced pulmonary vascular endothelial injury. Infect Immun 57: 1218–1226. 2925247 10.1128/iai.57.4.1218-1226.1989 PMC313253

[50] Puri RK, Travis WD, Rosenberg SA (1989) Decrease in interleukin 2-induced vascular leakage in the lungs of mice by administration of recombinant interleukin 1 alpha in vivo. Cancer Res 49: 969–976. 2783561

[51] Jonczyk MS, Simon M, Kumar S, Fernandes VE, Sylvius N, et al Genetic factors regulating lung vasculature and immune cell functions associate with resistance to pneumococcal infection. PLoS One 9: e89831 10.1371/journal.pone.0089831 24594938 PMC3940657

[52] Nichols JL, Gladwell W, Verhein KC, Cho HY, Wess J, et al Genome-wide association mapping of acute lung injury in neonatal inbred mice. FASEB J 28: 2538–2550. 10.1096/fj.13-247221 24571919 PMC4021442

[53] Lee SH, Girard S, Macina D, Busa M, Zafer A, et al (2001) Susceptibility to mouse cytomegalovirus is associated with deletion of an activating natural killer cell receptor of the C-type lectin superfamily. Nat Genet 28: 42–45. 11326273 10.1038/ng0501-42

[54] Scalzo AA, Fitzgerald NA, Simmons A, La Vista AB, Shellam GR (1990) Cmv–1, a genetic locus that controls murine cytomegalovirus replication in the spleen. J Exp Med 171: 1469–1483. 2159050 10.1084/jem.171.5.1469 PMC2187882

[55] Perera S, Holt MR, Mankoo BS, Gautel M Developmental regulation of MURF ubiquitin ligases and autophagy proteins nbr1, p62/SQSTM1 and LC3 during cardiac myofibril assembly and turnover. Dev Biol 351: 46–61. 10.1016/j.ydbio.2010.12.024 21185285 PMC3047806

[56] Sorokin L The impact of the extracellular matrix on inflammation. Nat Rev Immunol 10: 712–723. 10.1038/nri2852 20865019

[57] Wang T, Town T, Alexopoulou L, Anderson JF, Fikrig E, et al (2004) Toll-like receptor 3 mediates West Nile virus entry into the brain causing lethal encephalitis. Nat Med 10: 1366–1373. 15558055 10.1038/nm1140

[58] van Hinsbergh VW, van Nieuw Amerongen GP (2002) Intracellular signalling involved in modulating human endothelial barrier function. J Anat 200: 549–560. 12162723 10.1046/j.1469-7580.2002.00060.x PMC1570750

[59] Wessel F, Winderlich M, Holm M, Frye M, Rivera-Galdos R, et al Leukocyte extravasation and vascular permeability are each controlled in vivo by different tyrosine residues of VE-cadherin. Nat Immunol 15: 223–230. 10.1038/ni.2824 24487320

[60] Crawley SW, Shifrin DA Jr., Grega-Larson NE, McConnell RE, Benesh AE, et al (2014) Intestinal brush border assembly driven by protocadherin-based intermicrovillar adhesion. Cell 157: 433–446. 10.1016/j.cell.2014.01.067 24725409 PMC3992856

[61] Heemskerk N, van Rijssel J, van Buul JD Rho-GTPase signaling in leukocyte extravasation: An endothelial point of view. Cell Adh Migr 8: 67–75. 24621576 10.4161/cam.28244 PMC4049863

[62] Threadgill DW, Churchill GA (2012) Ten years of the collaborative cross. G3 (Bethesda) 2: 153–156. 22384393 10.1534/g3.111.001891 PMC3284322

[63] Kelada SN, Aylor DL, Peck BC, Ryan JF, Tavarez U, et al (2012) Genetic analysis of hematological parameters in incipient lines of the collaborative cross. G3 (Bethesda) 2: 157–165. 22384394 10.1534/g3.111.001776 PMC3284323

[64] Phillippi J, Xie Y, Miller DR, Bell TA, Zhang Z, et al (2014) Using the emerging Collaborative Cross to probe the immune system. Genes Immun 15: 38–46. 10.1038/gene.2013.59 24195963 PMC4004367

[65] Durrant C, Tayem H, Yalcin B, Cleak J, Goodstadt L, et al (2011) Collaborative Cross mice and their power to map host susceptibility to Aspergillus fumigatus infection. Genome Res 21: 1239–1248. 10.1101/gr.118786.110 21493779 PMC3149491

[66] Bottomly D, Ferris MT, Aicher LD, Rosenzweig E, Whitmore A, et al (2012) Expression quantitative trait Loci for extreme host response to influenza a in pre-collaborative cross mice. G3 (Bethesda) 2: 213–221. 22384400 10.1534/g3.111.001800 PMC3284329

[67] Rogala AR, Morgan AP, Christensen AM, Gooch TJ, Bell TA, et al (2014) The Collaborative Cross as a resource for modeling human disease: CC011/Unc, a new mouse model for spontaneous colitis. Mamm Genome 25: 95–108. 10.1007/s00335-013-9499-2 24487921 PMC3960486

[68] Rasmussen AL, Okumura A, Ferris MT, Green R, Feldmann F, et al (2014) Host genetic diversity enables Ebola hemorrhagic fever pathogenesis and resistance. Science. 10.1126/science.1259595 PMC4241145 25359852

[69] Mathes WF, Aylor DL, Miller DR, Churchill GA, Chesler EJ, et al (2011) Architecture of energy balance traits in emerging lines of the Collaborative Cross. Am J Physiol Endocrinol Metab 300: 124–134. 10.1152/ajpendo.00707.2010 PMC3118585 21427413

[70] Philip VM, Sokoloff G, Ackert-Bicknell CL, Striz M, Branstetter L, et al (2011) Genetic analysis in the Collaborative Cross breeding population. Genome Res 21: 1223–1238. 10.1101/gr.113886.110 21734011 PMC3149490

[71] Yount B, Roberts RS, Sims AC, Deming D, Frieman MB, et al (2005) Severe acute respiratory syndrome coronavirus group-specific open reading frames encode nonessential functions for replication in cell cultures and mice. J Virol 79: 14909–14922. 16282490 10.1128/JVI.79.23.14909-14922.2005 PMC1287583

[72] Yang H, Ding Y, Hutchins LN, Szatkiewicz J, Bell TA, et al (2009) A customized and versatile high-density genotyping array for the mouse. Nat Methods 6: 663–666. 10.1038/nmeth.1359 19668205 PMC2735580

[73] Mott R, Talbot CJ, Turri MG, Collins AC, Flint J (2000) A method for fine mapping quantitative trait loci in outbred animal stocks. Proc Natl Acad Sci U S A 97: 12649–12654. 11050180 10.1073/pnas.230304397 PMC18818

[74] Cox A, Ackert-Bicknell CL, Dumont BL, Ding Y, Bell JT, et al (2009) A new standard genetic map for the laboratory mouse. Genetics 182: 1335–1344. 10.1534/genetics.109.105486 19535546 PMC2728870

[75] Valdar W, Holmes CC, Mott R, Flint J (2009) Mapping in structured populations by resample model averaging. Genetics 182: 1263–1277. 10.1534/genetics.109.100727 19474203 PMC2728864

[76] Lopez-Romero P Agi4x44PreProcess: Preprocessing of Agilent 4x44 array data.

[77] Belisle SE, Tisoncik JR, Korth MJ, Carter VS, Proll SC, et al Genomic profiling of tumor necrosis factor alpha (TNF-alpha) receptor and interleukin–1 receptor knockout mice reveals a link between TNF-alpha signaling and increased severity of 1918 pandemic influenza virus infection. J Virol 84: 12576–12588. 10.1128/JVI.01310-10 20926563 PMC3004331

