# Supplementary figures and images for: Genome Wide Identification of SARS-CoV Susceptibility Loci Using the Collaborative Cross
Source: PLoS Genet. 2015 Oct 9;11(10):e1005504. doi: 10.1371/journal.pgen.1005504 (PMC4599853; doi:10.1371/journal.pgen.1005504)

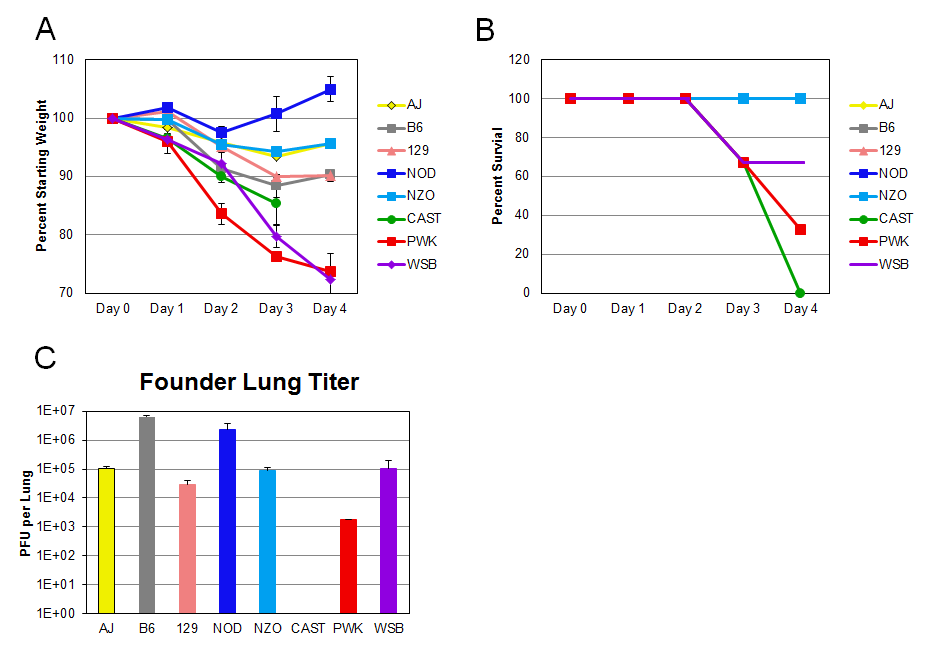

Supplement: S1 Fig — Weight loss (A), survival (B) and viral load (C) in the lung following infection with 105 PFU of SARS-CoV. (TIF) [file pgen.1005504.s001.tif]

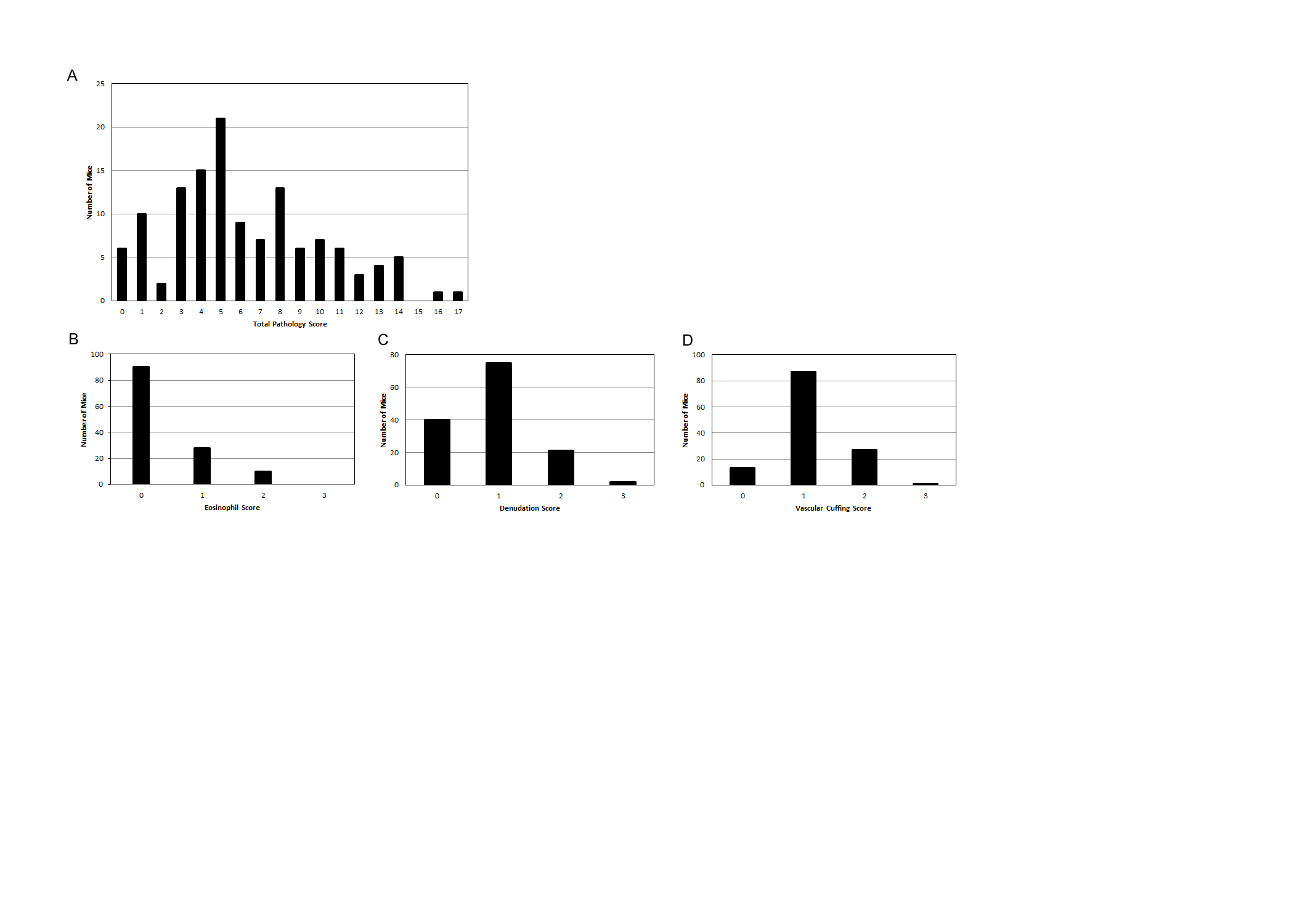

Supplement: S2 Fig — (A) Total pathology scores for each preCC line were summed and then grouped. (B) Breakdown of preCC lines by eosinophilia score. (C) Breakdown of preCC lines by airway denudation score. (D) Breakdown of preCC lines by vascular cuffing score. (TIF) [file pgen.1005504.s002.tif]

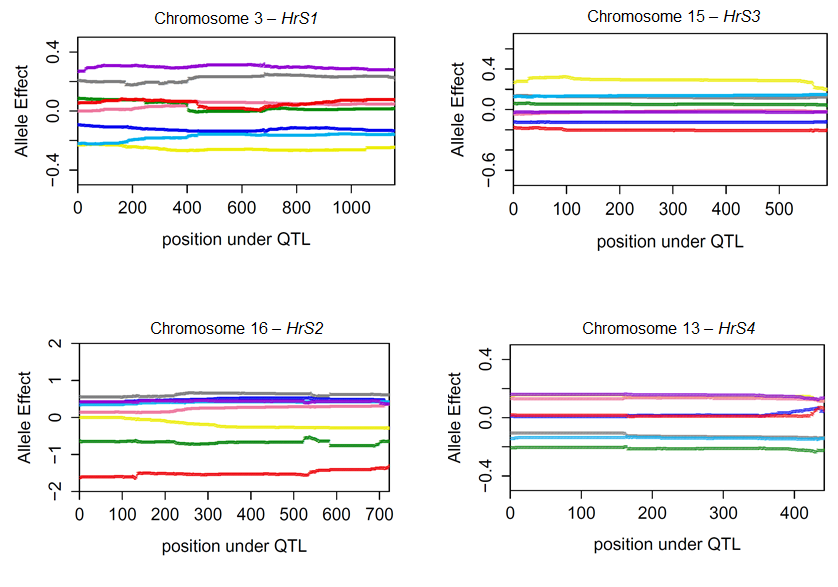

Supplement: S3 Fig — Effects of the founder alleles are shown for HrS1, HrS2, HrS3 and HrS4. The y-axis indicates the effect of a single founder allele in the QTL region on the overall phenotype. (TIF) [file pgen.1005504.s003.tif]
