# Supplementary material for: Genome Wide Identification of SARS-CoV Susceptibility Loci Using the Collaborative Cross
Source: PLoS Genet. 2015 Oct 9;11(10):e1005504. doi: 10.1371/journal.pgen.1005504 (PMC4599853; doi:10.1371/journal.pgen.1005504)
Supplement: S1 Table — LD50 was determined for each of the wild-derived founder strains used in the CC. (DOCX) [file pgen.1005504.s004.docx]

Table S1. Wild strain susceptibility.

| **Strain** | **LD50** |
| --- | --- |
| CAST/EiJ | 100-500 PFU |
| PWK/PhJ | 500-1000 PFU |
| WSB/EiJ | >1000 PFU |
