# Supplementary material for: Genome Wide Identification of SARS-CoV Susceptibility Loci Using the Collaborative Cross
Source: PLoS Genet. 2015 Oct 9;11(10):e1005504. doi: 10.1371/journal.pgen.1005504 (PMC4599853; doi:10.1371/journal.pgen.1005504)
Supplement: S3 Table — (DOCX) [file pgen.1005504.s006.docx]

| Table S3: Chr16 candidates | | |
| --- | --- | --- |
| Feature | Gene/ncRNA | Functional Variant? |
| Dlg1 | Gene | Y* |
| Mfi2 | Gene | Y |
| Gm15703 | Gene | N |
| Pigz | Gene | Y |
| Ncbp2 | Gene | Y |
| Senp5 | Gene | Y* |
| Pak2 | Gene | Y |
| Pigx | Gene | Y* |
| Cep19 | Gene | N |
| Gm15729 | Gene | Y |
| Lrrc33 | Gene | Y |
| Bex6 | Gene | Y |
| Fbxo45 | Gene | N |
| Wdr53 | Gene | N |
| Mir1946a | ncRNA | N |
| 2310010M20Rik | Gene | N |
| Rnf168 | Gene | Y |
| Metazoa_SRP | ncRNA | N |
| Ubxn7 | Gene | N |
| Tm4sf19 | Gene | Y |
| Tctex1d2 | Gene | N |
| Pcyt1a | Gene | N |
| Osta | Gene | Y |
| Zdhhc19 | Gene | N |
| Gm5405 | Gene | N |
| Tfrc | Gene | Y |
| Tnk2 | Gene | Y |
| Gm10818 | Gene | N |
| Muc4 | Gene | Y |
| Muc20 | Gene | Y |
| 1700021K19Rik | Gene | Y |
| Fyttd1 | Gene | N |
| Lrch3 | Gene | Y* |
| Iqcg | Gene | Y |
| Lmln | Gene | Y |
| RNAseP_nuc | ncRNA | N |
| Osbpl11 | Gene | Y |
| Snx4 | Gene | Y |
| Metazoa_SRP | ncRNA | N |
| Zfp148 | Gene | Y |
| Slc12a8 | Gene | Y |
| Heg1 | Gene | Y |
| Gm15658 | Gene | Y |
| Gm15657 | Gene | N |
| Muc13 | Gene | Y |
| Itgb5 | Gene | Y |
| Umps | Gene | Y* |
| Gm15829 | Gene | N |
| Kalrn | Gene | Y |
| Ropn1 | Gene | N |
| Ccdc14 | Gene | Y |
| Mylk | Gene | Y |
| Ptplb | Gene | N |
| Adcy5 | Gene | N |
| Sec22a | Gene | Y |
| SNORA73 | ncRNA | N |
| U1 | ncRNA | N |
| Pdia5 | Gene | Y |
| Sem5b | Gene | N |
| Dirc2 | Gene | Y* |
| Hspbap1 | Gene | Y |
| Parp14 | Gene | Y |
| Gm10237 | Gene | N |
| Dtx3l | Gene | N |
| Parp9 | Gene | Y* |
| Gm15564 | Gene | Y |
| Kpna1 | Gene | Y* |
| Wdr5b | Gene | N |
| Fam162a | Gene | N |
| Ccdc58 | Gene | N |
| Csta | Gene | N |
| Gm10092 | Gene | N |
| U6 | ncRNA | N |
| Gm1975 | Gene | Y |
| BC100530 | Gene | Y* |
| Gm15845 | Gene | N |
| Stfa2 | Gene | Y |
| U6 | ncRNA | N |
| Stfa3 | Gene | Y |
| Casr | Gene | Y |
| Cd86 | Gene | Y |
| Ildr1 | Gene | Y |
| * Transcript has private nonsense mediated decay SNPs, but other alleles also have other nonsense mediated decay SNPs | | |
