# Supplementary material for: Genome Wide Identification of SARS-CoV Susceptibility Loci Using the Collaborative Cross
Source: PLoS Genet. 2015 Oct 9;11(10):e1005504. doi: 10.1371/journal.pgen.1005504 (PMC4599853; doi:10.1371/journal.pgen.1005504)
Supplement: S4 Table — (DOCX) [file pgen.1005504.s007.docx]

| Table S4: Chr15 Candidates | | |
| --- | --- | --- |
| Feature | Gene/ncRNA | Functional Variant? |
| Kcnk9 | Gene | N |
| Trappc9 | Gene | N |
| Eif2c2 | Gene | N |
| Ptk2 | Gene | N |
| Gpr20 | Gene | N |
| Bai1 | Gene | Y |
| 1700016M24Rik | Gene | N |
| Arc | Gene | N |
| Jrk | Gene | N |
| Ly6d | Gene | N |
| Cyp11b1 | Gene | N |
| Cyp11b2 | Gene | N |
| 2010109I03Rik | Gene | N |
| Ly6e | Gene | N |
| Ly6i | Gene | N |
| Ly6a | Gene | N |
| Ly6c1 | Gene | N |
| Ly6c2 | Gene | N |
| Ly6g | Gene | N |
| BC025446 | Gene | N |
| Gm10238 | Gene | N |
| Ly6f | Gene | N |
| 9030619P08Rik | Gene | N |
| Gm20654 | Gene | N |
| Zc3h3 | Gene | N |
| * Transcript has private nonsense mediated decay SNPs, but other alleles also have other nonsense mediated decay SNPs | | |
