# Supplementary material for: Genome Wide Identification of SARS-CoV Susceptibility Loci Using the Collaborative Cross
Source: PLoS Genet. 2015 Oct 9;11(10):e1005504. doi: 10.1371/journal.pgen.1005504 (PMC4599853; doi:10.1371/journal.pgen.1005504)
Supplement: S5 Table — (DOCX) [file pgen.1005504.s008.docx]

| Table S5: HrS4 Candidates | | |
| --- | --- | --- |
| Feature | Gene/ncRNA | Functional Variant? |
| Auh | Gene | Y* |
| n-R5s54 | ncRNA | N |
| Nfil3 | Gene | N |
| Ror2 | Gene | N |
| Sptlc1 | Gene | N |
| Msx2 | Gene | N |
| Gm5449 | Gene | N |
| Drd1a | Gene | N |
| Sfxn1 | Gene | N |
| Hrh2 | Gene | N |
| Gm16578 | Gene | N |
| Cplx2 | Gene | N |
| Gm16248 | Gene | N |
| 7Sk | ncRNA | N |
| Thoc3 | Gene | N |
| Gm2830 | Gene | N |
| 4732471D19Rik | Gene | Y* |
| 4833439L19Rik | Gene | Y |
| Arl10 | Gene | N |
| AC155262.1 | ncRNA | Y |
| Nop16 | Gene | Y* |
| Higd2a | Gene | Y |
| Cltb | Gene | N |
| Faf2 | Gene | Y* |
| Rnf44 | Gene | N |
| Cdhr2 | Gene | Y |
| Gprin1 | Gene | Y |
| Sncb | Gene | N |
| Gm16249 | Gene | N |
| Eif4e1b | Gene | Y* |
| Tspan17 | Gene | N |
| Gm16250 | Gene | N |
| * Transcript has private nonsense mediated decay SNPs, but other alleles also have other nonsense mediated decay SNPs | | |
